# Spin torque control of antiferromagnetic moments in NiO

**DOI:** 10.1038/s41598-018-32508-w

**Published:** 2018-09-21

**Authors:** Takahiro Moriyama, Kent Oda, Takuo Ohkochi, Motoi Kimata, Teruo Ono

**Affiliations:** 1 Institute for Chemical Research, Kyoto University, Uji, Kyoto 611-0011 Japan; 2 Center for Spintronics Research Network, Osaka University, Toyonaka, Osaka 560-8531 Japan; 3 Japan Synchrotron Radiation Research Institute, Sayo, Hyogo 679-5198 Japan; 4 Institute for Materials Research, Tohoku University, Sendai, Miyagi 980-8577 Japan

## Abstract

For a long time, there were no efficient ways of controlling antiferromagnets. Quite a strong magnetic field was required to manipulate the magnetic moments because of a high molecular field and a small magnetic susceptibility. It was also difficult to detect the orientation of the magnetic moments since the net magnetic moment is effectively zero. For these reasons, research on antiferromagnets has not been progressed as drastically as that on ferromagnets which are the main materials in modern spintronic devices. Here we show that the magnetic moments in NiO, a typical natural antiferromagnet, can indeed be controlled by the spin torque with a relatively small electric current density (~4 × 10^7^ A/cm^2^) and their orientation is detected by the transverse resistance resulting from the spin Hall magnetoresistance. The demonstrated techniques of controlling and detecting antiferromagnets would outstandingly promote the methodologies in the recently emerged “antiferromagnetic spintronics”. Furthermore, our results essentially lead to a spin torque antiferromagnetic memory.

## Introduction

The majority of spintronics research and applications has so far dealt with ferromagnetism, with much less attention given to antiferromagnetic materials. Although they have no net magnetic moment, the microscopic magnetic moments in antiferromagnetic materials can in principle exhibit similar spintronic effects, such as various magnetoresistance effects and the spin torque effect^1^, as seen in ferromagnetic materials^2^.

Theoretical and experimental studies have suggested that it is possible to control the antiferromagnetic moments by the spin transfer torque due to a consequence of the interaction between the local moment and the itinerant electron spins^3–7^. It is also predicted and demonstrated that magnetoresistances, such as anisotropic magnetoresistance^8–11^, the planer Hall effect^8,11^, and the spin Hall magnetoresistance^12–14^, were available for detecting the orientation of the magnetic moments in antiferromagnets.

Wadley *et al*.^8^ reported that the CuMnAs having a broken inversion symmetry in its spin sublattices gives rise to the relativistic effective fields by a flow of an electric current in itself. This effective field switches the magnetic moments and the orientation is read by the magnetoresistance. Although the seminal reports^8,15–17^ seem to magnificently advance a spin operation of the antiferromagnet using this relativistic effective field, choice of the materials having such a complex unit cell structure may be limited. To further pave a wide pathway of antiferromagnetic spintronics, it is desirable to seek the control of more generic antiferromagnetic materials.

In this letter, we show a control of the orientation of the magnetic moments in an antiferromagnetic NiO by the spin Hall effect induced spin transfer and also show an electrical detection of the orientation of the magnetic moments based on spin Hall magnetoresistance (SMR). Our results essentially demonstrated a spin torque antiferromagnetic memory with NiO.

NiO is one of the most common natural antiferromagnetic oxides the study of which dates back to the dawn of the antiferromagnetism^18^. It still continues to be an archetype material for investigating interesting novel phenomena, such as the THz magnon dynamics^19^, the spin current transmission^20,21^, and the spin Hall magnetoresistance^12–14^. NiO has a rock-salt structure with magnetic moments inhabiting on Ni cations. The magnetic moments lie in one of the {111} planes and are ferromagnetically aligned on an {111} plane and they antiferromagnetically couple with the magnetic moments in neighboring {111} planes by super exchange coupling^18^.

**Figure 1:**
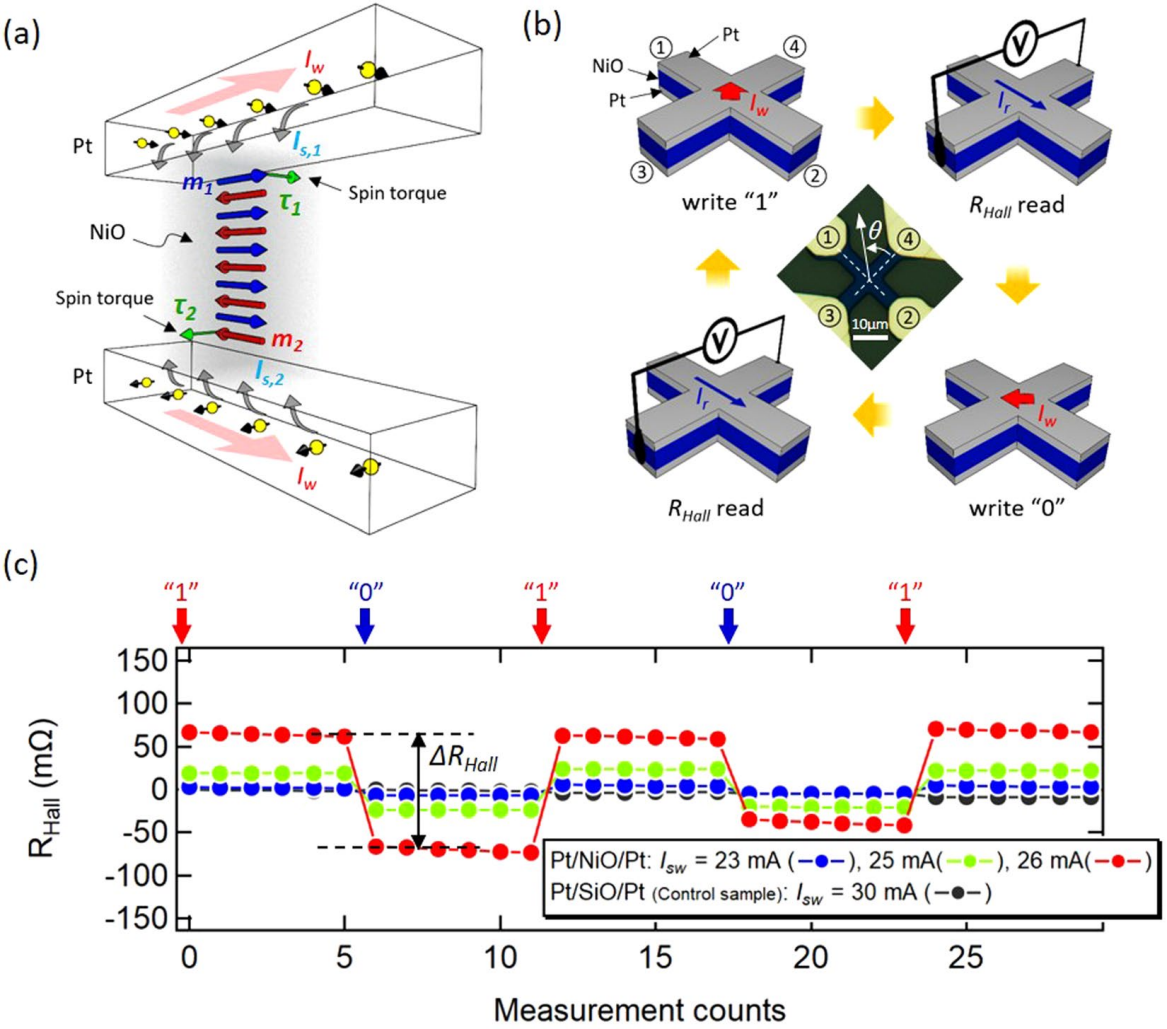
The spin torque writing scheme and the sequential write-read memory operation. (**a**) The basic principle of the spin torque rotation of the antiferromagnets using the spin Hall effect in a Pt/NiO/Pt multilayer structure. The writing current *I*_*w*_ in the Pt layers invokes the spin Hall effect, and injects the spin currents with the spin polarization ***I***_***s,1***_ and ***I***_***s,2***_ toward the antiferromagnetic NiO. The spin currents exert a spin torque ***τ***_***1***_ and ***τ***_***2***_ (green arrows) on the microscopic magnetic moments in the same rotation direction. (**b**) Measurement procedure of the spin torque write and the Hall resistance read. *R*_*Hall*_ is measured after each write “1” and “0”. Microscope image of the Hall cross structure is shown in the center. Our common definition of the angle *θ* is also shown. (**c**) The sequential write-read operation in Pt/NiO 10 nm/Pt with *I*_*w*_ = 23, 25, and 26 mA and Pt/SiO_2_ 10 nm/Pt with *I*_*w*_ = 30 mA. The arrows on the top represent the write “1” and “0” operations described in (b). As indicated in the graph, *∆R*_*Hall*_ is defined as *∆R*_*Hall*_ = *R*_*Hall*_ (“1”) − *R*_*Hall*_ (“0”) where *R*_*Hall*_ (“1”) and *R*_*Hall*_ (“0”) respectively represent the *R*_*Hall*_ after the write “1” and the write “0”.

Layer structures employed in this study are epitaxially grown Pt 4 nm/NiO 10 nm/Pt 4 nm formed on MgO (111) single crystal substrate by magnetron sputtering. The reflection high-energy electron diffraction (RHEED) analyses confirmed that the each layer was grown as single crystal with (111) face as expected by the lattice matching of the MgO, NiO and Pt (see Methods and Supplementary Information (SI) for details.). Figure 1(a) shows the basic principle of the spin torque rotation of the antiferromagnetic moments in a Pt/NiO/Pt multilayer structure. A current flow ***I***_***w***_ in Pt layers invoke the spin Hall effect^22,23^ and injects a spin current with a spin polarization ***I***_***s***_ into the NiO layer by ${{\boldsymbol{I}}}_{{\boldsymbol{s}}}=(\hslash /2e){\theta }_{SH}{{\boldsymbol{I}}}_{{\boldsymbol{w}}}\times {\boldsymbol{q}}$ where $\hslash$ is the reduced planck constant, *e* is the elementary charge, *θ*_*SH*_ is the spin Hall angle, and ***q*** is the unit vector parallel to the flow of the spin current. The spin current with the spin polarization ***I***_***s,1***_ from the top Pt layer exerts a spin torque ***τ***_***1***_ on the magnetic moment ***m***_***1***_ in the NiO layer as **τ**_**1**_ ∝ ***m***_**1**_ × ***I***_***s*,1** _× ***m***_**1**_. The spin current from the bottom Pt layer similarly results in a spin torque **τ**_**2**_ ∝ ***m***_**2**_ × ***I***_***s*,2**_ × ***m***_**2**_. Assuming the antiferromagnetic order is coherent in the thickness direction and also assuming a situation where two sets of the spin torque act on the bipartite magnetic moments in the same rotation direction as depicted in Fig. 1(a), the magnetic moments can efficiently rotate without a cost to increasing the exchange energy (or to competing with the molecular field) which strongly ties the neighboring moments. The magnetic moments rotate until they become orthogonal to the current flow and no more spin torque is exerted (See also the macro-spin simulations in Section 2 of SI). In order to demonstrate the control of the antiferromagnetic moments, we used the Hall bar structure with an experimental procedure described in Fig. 1(b). A writing current *I*_*w*_ flowing from the electrode 2 and 3 to the electrodes 1 and 4, as represented by write “1”, rotates the magnetic moments and stabilizes them orthogonal to the direction of *I*_*w*_. In the same manner, another writing current *I*_*w*_ flowing from the electrode 2 and 4 to the electrode 1 and 3 writes “0”. The orientation of the magnetic moments is read, after each write, by the transverse resistance (*R*_*Hall*_) with a small excitation current *I*_*r*_ flowing from the electrode 1 to 2. We particularly took advantage of SMR to read out the orientation of the magnetic moments. In the phenomenology of SMR^24^, the longitudinal and transverse resistance vary by the relative angle between the magnetic moments in a magnetic layer and the spin polarization of the spin current created by the spin Hall effect in a non-magnetic layer. By adopting the equation for the ferromagnetic case^24^ for our geometry, *R*_*Hall*_ due to the orientation of the NiO magnetic moments can be written as,

1 $${R}_{Hall}\propto -{\rm{\Delta }}{R}_{SMR}\,\sin \,{\theta }_{n}\,\cos \,{\theta }_{n},$$

where Δ*R*_*SMR*_ is the coefficient of SMR and *θ*_*n*_ is the angle of the Néel vector ***n*** with ***n*** = ***m***_**1**_−***m***_**2**_ (see Fig. 1(b) for our circular coordinate with the definition of the general angle *θ*.). ***m***_**1**_ and ***m***_**2**_ represent a unit vector for the bipartite magnetic moments as depicted in Fig. 1(a). Here we only consider the in-plane orientation of the magnetic moments.

Figure 1(c) shows representative results of the sequential write-read operation in Pt 4 nm/NiO 10 nm/Pt 4 nm and Pt 4 nm/SiO_2_ 10 nm/Pt 4 nm (a control sample) with various *I*_*w*_. The operation of write “1” results in a high resistance state and “0” in a low state, which is coherently explained by the spin torque rotation of the magnetic moments and the change of *R*_*Hall*_ due to SMR described in Eq. (1). For instance, with the write “1”, the spin torque directs the Neel vector at *θ*_*n*_ = 135° or 315° (these two possibilities are degenerated in our experiment.) as the magnetic moments ***m***_**1**_ and ***m***_**2**_ become orthogonal to the direction of *I*_*w*_. Equation (1) yields a high resistance state with *θ*_*n*_ = 135° or 315° after write “1” and a low state with *θ*_*n*_ = 45° or 225° after write “0”. One can also clearly see the *ΔR*_*Hall*_, which is defined as the change of *R*_*Hall*_ after each writing operation, varies with *I*_*w*_. The control experiments in Pt/SiO_2_/Pt, where SiO_2_ is a diamagnetic material, show no *ΔR*_*Hall*_. We note that the results of the write-read operations are found irrelevant to the polarity of *I*_*w*_.

**Figure 2:**
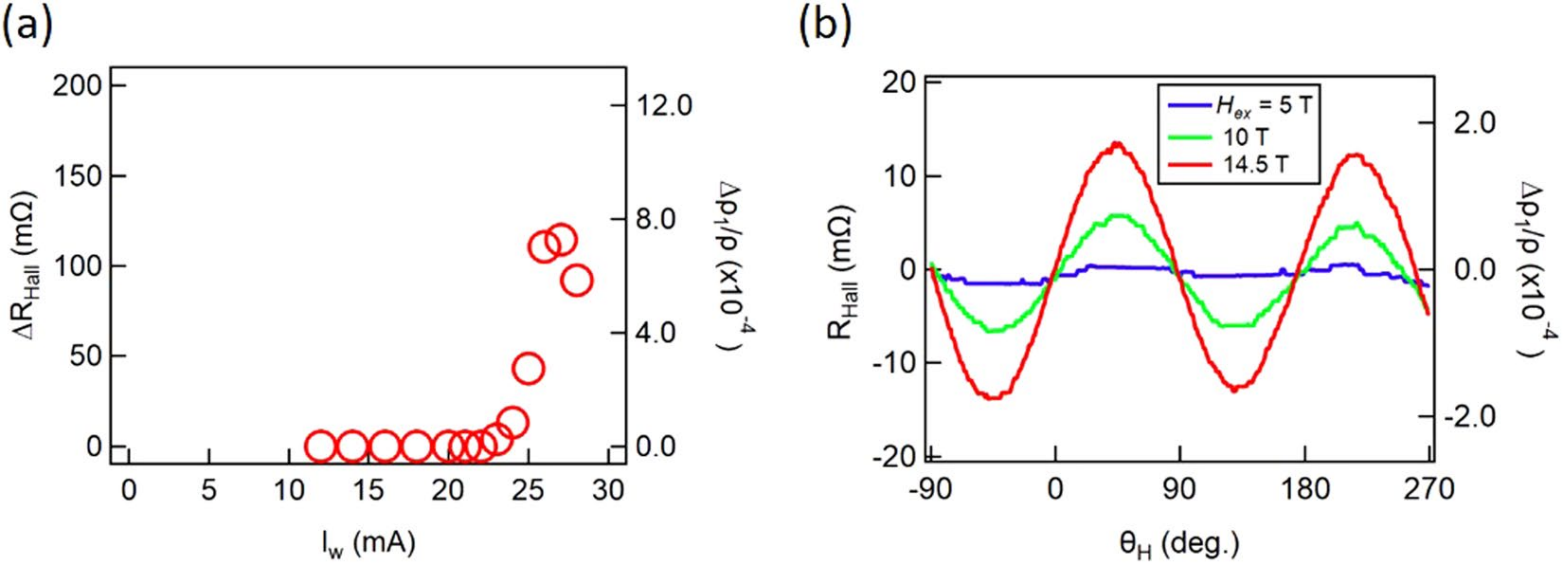
Writability by the spin torque and robustness against an external field. (**a**) *∆R*_*Hall*_ and Δ*ρ*_1_/*ρ* as a function of the writing current *I*_*w*_ for Pt/NiO 10 nm/Pt. (**b**) *R*_*Hall*_ measurements for Pt/NiO 10 nm/Pt in a rotating magnetic field *H*_*ex*_ = 5, 10, and 14.5 Tesla. *θ*_*H*_ is the field angle in the circular coordinate shown in Fig. 1(b).

Figure 2(a) shows *I*_*w*_ dependence of *ΔR*_*Hall*_ for the Pt/NiO/Pt. We observed a threshold around 20 mA, corresponding to the current density of 4 × 10^7^ A/cm^2^. The transverse resistivity change Δ*ρ*_1_ over the resistivity *ρ* of Pt (Δ*ρ*_1_/*ρ*) was translated from *ΔR*_*Hall*_ (See SI for the derivation.). The maximum *ΔR*_*Hall*_ = 112 mΩ corresponding to Δ*ρ*_1_/*ρ* = 7.1 × 10^−4^ was obtained with *I*_*w*_ = 27 mA. On the other hand, *R*_*Hall*_ in a rotating magnetic field with the angle *θ*_*H*_, as shown in Fig. 2(b), shows a sin2*θ*_*H*_ dependence as expected by the SMR due to the rotation of the spin-flopped Neel vector. We obtained Δ*ρ*_1_/*ρ *= 1.5 × 10^−4^ with 14.5 T and it seems to be still increasing with increasing the field magnitude^14^. Both Δ*ρ*_1_/*ρ* obtained by the two different measurements are within a reasonable range comparing to previously reported values^13,14^. We emphasize that the spin torque switching using our scheme requires the current density of only ~10^7^ A/cm^2^ to control the Neel vectors while the external field requires more than 5 T.

In order for our spin torque writing scheme (Fig. 1(a)) to work, the minimum requirement may be that the magnetic moments of the NiO need to lie in the sample plane and the staggered magnetic moments are coherent in the thickness direction. This condition can be satisfied as the NiO layer is epitaxially grown with (111) plane on which the magnetic moments can lie although magnetic moments laying on other diagonals of {111} may not contribute to the switching. One may also notice that our writing scheme requires the NiO layer to have oppositely pointing magnetic moments at the top and bottom interfaces as exactly depicted in Fig. 1(a) (Otherwise, two sets of the spin torque compete with each other.). Considering the chance of being such a spin configuration, we expect that some parts of the device area are suitable for the writing scheme.

**Figure 3:**
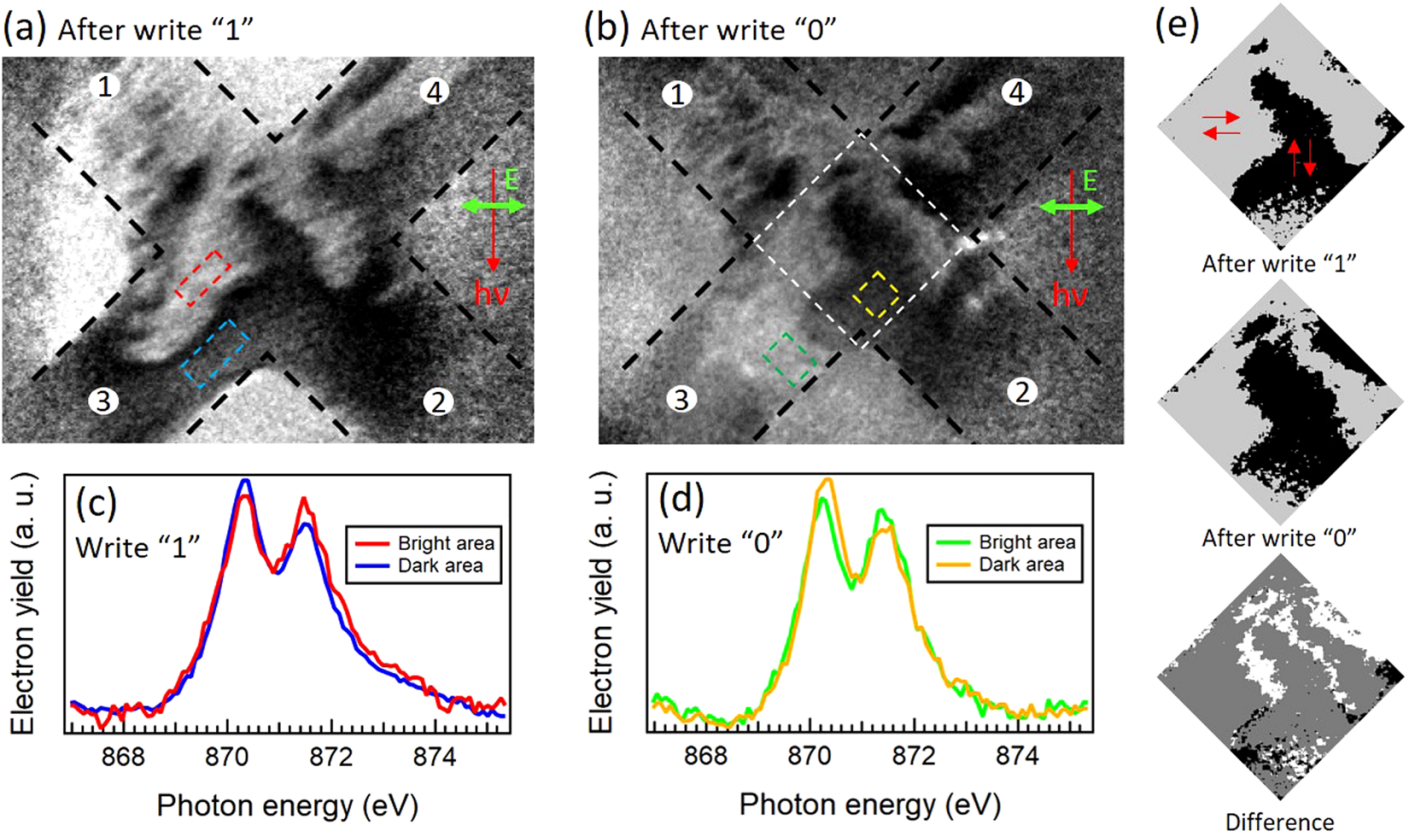
The NiO domains after the spin-torque writing with X-ray magnetic linear dichroism-photoemission electron microscopy (XMLD-PEEM). XMLD-PEEM images, processed by dividing the higher energy peak by the lower energy peak at the Ni-L2 edge, after the write “1” (**a**) and the write “0”. (**b**) The black dotted lines indicate the Hall bar structure. The incident and linear-polarized direction of the X-ray beam are indicated with the red and green arrows, respectively. The rectangles show the region where the NiO domain was modified. (**c**) and (**d**) are electron yield as a function of the photon energy scan of the rectangle regions. (**e**) The post-processed monochrome images from the center region framed by the white square in (**b**) show the delimiting boundaries of the NiO domains (See SI for more details). Difference between the state “1” and “0” is also shown.

Figure 3 shows the results of the NiO antiferromagnetic domain imaging of the epitaxial Pt 4 nm/NiO 10 nm/Pt 4 nm sample after the write “0” and “1” operations observed by X-ray magnetic linear dichroism-photoemission electron microscopy (XMLD-PEEM) in BL17SU RIKEN beamline at SPring-8^25^. NiO antiferromagnetic spin domains (S-domains) were observed at the Ni-L_2_ edge in a similar manner described in Refs^26^ and^27^. The images after the write “0” and “1” shown in Fig. 3(a,b) were processed by dividing the higher energy peak by the lower energy peak at the L_2_ edge indicated in Fig. 3(c,d). Therefore, the image contrast directly indicates the NiO S-domains. By the incident direction of the linearly polarized X-ray beam (indicated in Fig. 3(a,b)), the light (dark) regions reflect the Neel vector oriented perpendicular (collinear) to the beam incidence. The NiO domain patterns after the write “0” and “1” clearly differ. They remarkably image the partial rotation of the Neel vector within the Hall bar structure as viewed in the Fig. 3(a,b). For instance, after the write “0”, the black region indicated by the yellow rectangle is widen and the white region indicated by the green rectangle appears. The post-processed monochrome images around the central area of the Hall cross shown in Fig. 3(e) clearly resolve the expansion of the NiO domain after the writing operation. These final states of the Neel vector are perpendicular to the switching current flow, which is consistent with our proposed model described in Fig. 1(a). It is important to emphasize that the Neel vector rotation is not coherent rotation over the Hall bar structure but it occurs in a part satisfying the spin configuration as discussed above.

Since the size of the NiO antiferromagnetic domains is comparable to the Hall bar and the locations where the domain rotates are random as shown in Fig. 3, one may expect that the switching characteristics can easily vary from sample to sample. We indeed found that *ΔR*_*Hall*_ and Δ*ρ*_1_/*ρ* with the same *I*_*w*_ are quite scattered from sample to sample as shown in Table 1. For possible antiferromagnetic memory applications in which a full rotation of the NiO domains would be desirable, further efforts may be required to increase the area of switching by more rigorously controlling the appropriate spin configurations in the NiO layer as well as at the NiO/Pt interfaces. Challenging but realistic approaches would be an atomic control of the NiO layer stack and introduction of an additional anisotropy to remove the degeneracy of the NiO {111} easy planes.

**Table 1:** *ΔR*_*Hall*_ and Δ*ρ*_1_/*ρ* with *I*_*w*_ = 26 mA for four different samples. Standard deviation (SD.) is shown in the last column.

Sample^#^	1	2	3	4	SD.
*ΔR*_*Hall*_ (mΩ)	16.1	110.4	39.6	6.9	46.8
Δ*ρ*_1_/*ρ* × 10^−4^	1.0	6.9	2.5	0.4	2.9

In summary, we successfully demonstrated the spin-torque control and the resistive read of the magnetic moments in NiO which is an archetype collinear antiferromagnet. The scheme of controlling and detecting antiferromagnets presented here can be applicable to a wide variety of collinear antiferromagnets, and perhaps other types of antiferromagnets. We emphasize that the demonstrated spin-torque control of NiO is apparently much more efficient than a field control requiring >5 Tesla. The detection scheme using SMR is much more easily accessible than traditionally used neutron scattering^18^ as well as X-ray magnetic linear dichroism^28^. Thus, those of our easily accessible combined methodologies will open up more fundamental research opportunities on antiferromagnets. Ultimately, we stress that basic requirements for practical antiferromagnetic spintronic devices, i.e. the electrical control and detection of antiferromagnetic moments, are now fulfilled.

## Methods

### Sample fabrication and characterization

The multilayers were formed by magnetron sputtering on MgO (111) single crystal substrate. We used a sintered NiO sputtering target for the deposition of the NiO layer. All the depositions were done at room temperature with a base pressure below 2 × 10^−6^ Pa. The reflection high-energy electron diffraction (RHEED) was performed *in-situ* after the deposition of each layer. The control samples Pt 4 nm/SiO 10 nm/Pt 4 nm were formed on a thermally oxidized Si substrate by magnetron sputtering. As shown in Fig. 1(b), the Hall bar structure with 5 µm-wide channel was patterned by a conventional photolithography followed by an Ar ion milling. Ti (5 nm)/Au (100 nm) was deposited for the contact pads. All the measurements were performed at room temperature.

### The spin torque write-read procedure

We used a multiplexer system built with Keithley 2700 to quickly route the current flow and voltage probes among the electrodes 1~4. We applied *I*_*w*_ for 3 seconds to write, then shut it off, and started reading *R*_*Hall*_ with an excitation current *I*_*r*_ of 1 mA. *R*_*Hall*_ was read multiple times with an interval of 30 seconds.

### XMLD-PEEM imaging

XMLD-PEEM was performed in the beamline BL17SU at SPring-8 capable of generating linearly polarized X-ray beams with the photon energy ranging from 500 to 900 eV. An *s*-polarized (i.e. the oscillating electric field is within the sample plane) X-ray beams were incident to the sample with an incident angle of 16° with respect to the sample surface. The spin torque writing operations were performed outside the PEEM vacuum chamber. After each writing operation, the sample was introduced in the PEEM chamber and observed the images.

## Electronic supplementary material

Supplementary Information
