# Supplementary material for: Spin torque control of antiferromagnetic moments in NiO
Source: Sci Rep. 2018 Sep 21;8:14167. doi: 10.1038/s41598-018-32508-w (PMC6155024; doi:10.1038/s41598-018-32508-w)
Supplement: Supplementary file 1 — Supplementary Information [file 41598_2018_32508_MOESM1_ESM.docx]

**Spin torque control of antiferromagnetic moments in NiO**

Takahiro Moriyama^1,2^, Kent Oda^1^, Takuo Ohkochi^3^, Motoi Kimata^4^, and Teruo Ono^1,2^

*^1^ Institute for Chemical Research, Kyoto University, Uji, Kyoto, 611-0011, Japan*

*^2^Center for Spintronics Research Network, Osaka University, Toyonaka, Osaka, 560-8531, Japan*

*^3^ Japan Synchrotron Radiation Research Institute, Sayo, Hyogo, 679-5198, Japan*

*^4^Institute for Materials Research, Tohoku University, Sendai, Miyagi, 980-8577, Japan*

Supplementary Information

1. **Epitaxy of the Pt/NiO/Pt on MgO (111)**

Figures S1 show the reflection high-energy electron diffraction (RHEED) images for each interface which clearly indicate the coherent crystal orientation throughout the structure. Rotational symmetry of the diffraction patterns suggests that the each layer is single crystal and (111)-orientated as expected by the lattice matching of the MgO, NiO, and Pt crystal lattices.


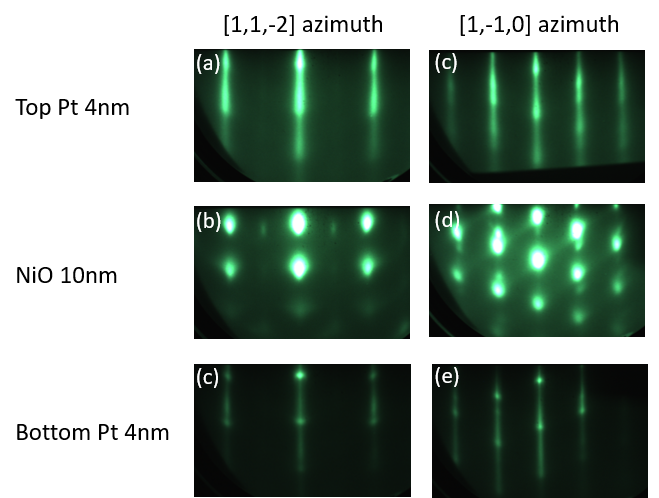


**Figure S1 Epitaxy of the Pt 4nm/ NiO 10nm/ Pt 4nm grown on MgO (111).** Reflection high-energy electron diffraction (RHEED) images for the surface of (a)(b) MgO(111)/Pt 4 nm, (c)(d) MgO(111)/Pt 4/NiO 10 nm, and (e)(f) MgO(111)/Pt 4/NiO 10/ Pt 4nm, observed in the [1,1,-2] and [1,-1,0] azimuths as indicated in the figures.

1. **Macro-spin simulations of the spin torque control**

Our spin torque operation of the antiferromagnetic NiO is modeled by the Landau–Lifshitz–Gilbert (LLG) equation implemented with the spin current injection by the spin Hall effect. For the simplicity, we assume the two macro-spin sublattices represented by the unit vectors **m_1_** and **m_2_**. The dynamics of each sublattice is described by the LLG equation with the spin torque term as,

$$\frac{d\mathbf{m}_{\boldsymbol{\sigma}}}{dt}=-\gamma\mathbf{m}_{\boldsymbol{\sigma}}\times\mathbf{H}_{\boldsymbol{\sigma}}+\gamma\alpha\mathbf{m}_{\boldsymbol{\sigma}}\times\mathbf{H}_{\boldsymbol{\sigma}}\times\mathbf{m}_{\boldsymbol{\sigma}}\mathbf{+}\gamma/\left( M_{\sigma}t \right)\mathbf{m}_{\boldsymbol{\sigma}}\times\mathbf{J}_{\boldsymbol{s,\sigma}}\times\mathbf{m}_{\boldsymbol{\sigma}}\boldsymbol{,} (S1)$$

where σ corresponds to the sublattice (σ = 1 or 2), and $\gamma$, $\alpha$, $M_{\sigma}$ *t* are the gyromagnetic ratio, Gilbert damping constant, magnetic moment per sublattice volume, and thickness of the NiO. $\mathbf{J}_{\boldsymbol{s,\sigma}}\boldsymbol{=}\left( \hbar/{2e} \right)\theta_{SH}\mathbf{J}_{\mathbf{c}}\boldsymbol{\times}\mathbf{q}_{\boldsymbol{\sigma}}$ is the polarization of the spin current injected by the spin Hall effect where $\boldsymbol{\hbar}$ is the Planck’s constant, *e* the elementary charge, $\theta_{SH}$the spin Hall angle, $\mathbf{J}_{\mathbf{c}}$ the charge current density, $\mathbf{q}_{\boldsymbol{\sigma}}$ the flow direction of the spin current. The effective field $\mathbf{H}_{\boldsymbol{\sigma}}$ applied on the sublattices can be written as,

$$\mathbf{H}_{\mathbf{1}}\boldsymbol{=}\mathbf{H}_{\mathbf{0}}\boldsymbol{-}H_{e}\mathbf{m}_{\mathbf{2}}\boldsymbol{,}(S2)$$

$$\mathbf{H}_{\mathbf{2}}\boldsymbol{=}\mathbf{H}_{\mathbf{0}}\boldsymbol{-}H_{e}\mathbf{m}_{\mathbf{1}}\boldsymbol{,}(S3)$$

where $\mathbf{H}_{\boldsymbol{0}}\mathbf{=}\mathbf{H}_{\boldsymbol{ext}}\boldsymbol{+}\mathbf{H}_{\boldsymbol{a}}$ with the anisotropy field $\mathbf{H}_{\boldsymbol{a}}$and the external applied field $\mathbf{H}_{\boldsymbol{ext}}$, and $H_{e}$ is the exchange field which couples **m_1_** and **m_2_**. $H_{e}>0$ is set for the antiferromagnetic coupling.

We presume that the easy plane of the NiO is in the x-y plane. Fig. S2 models the Pt/NiO/Pt structure where the spin current due to the spin Hall effect by the charge current $\mathbf{J}_{\mathbf{c}}\boldsymbol{\parallel}\hat{\mathbf{x}}$ is injected in both $\mathbf{q}_{\mathbf{1}}$= $-\hat{\mathbf{z}}$ and $\mathbf{q}_{\mathbf{2}}$= $+\hat{\mathbf{z}}$ directions.

We assume the anisotropy energy$E_{a}=K_{1}\sin^{2} \theta+K_{2}\sin^{4} \theta+K_{3}\sin^{6} \theta\cos6\varphi$, where $\theta$ and $\varphi$ are respectively the polar angle and the azimuth angle of **m_1_** and **m_2_**, in order to take into account the easy plane anisotropy in (111) plane and the 6-fold magnetic anisotropy within the easy plane^[[1]](#endnote-1)^. We set the parameters shown in Table S1 to simulate the spin torque control of the NiO. We note that our macro-spin simulations totally neglect the spin current gradient applied on the Neel vectors inside the NiO, which could be the case in our real samples. Whether the spin current gradient is critical in this case is controversial since the spin angular momentum carried by the electron should be given to the first monolayer of the magnetic system because electrons cannot diffuse into the insulator. Spin currents propagation carried by the spin waves deep into the insulator^[[2]](#endnote-2),^^[[3]](#endnote-3)^ is not likely because, in the present experimental situation, the spin current from the Pt does not excite the spin waves rather triggers the rotation of the Neel vector. As we will show below, nevertheless, we believe that this macro-spin simulations represents the characteristic of our spin torque control of the NiO.

Figure S3 shows a typical trajectory of **m_1_** and **m_2_** when they rotate by the spin torque for the case of the Pt/NiO/Pt structure. The writing current $\mathbf{J}_{\mathbf{c}}$ is applied for a duration of 10 ns as shown in Fig. S3 (a). **m_1_** and **m_2_** rotate from the initial state (**m_1_** = $+\hat{\mathbf{x}}$, **m_2_** = $-\hat{\mathbf{x}}$) to one of the easy axes (**m_1_** = ${\sqrt{3}x}/2\hat{\mathbf{x}}{+1}/2\hat{\mathbf{y}}$, **m_2_** = ${-\sqrt{3}x}/2\hat{\mathbf{x}}{-1}/2\hat{\mathbf{y}}$). The critical current for the rotational switching over the 6-fold magnetic anisotropy ${\mathbf{J}_{\mathbf{sw}}}/{H_{a\varphi}}$ as a function of the exchange field $H_{e}$ is shown in Fig. S4 (a). We adopted ${\mathbf{J}_{\mathbf{sw}}}/{H_{a\varphi}}$ here since the $\mathbf{J}_{\mathbf{sw}}$ is found to be linearly proportional to $H_{a\varphi}$ irrespective of $H_{e}$ as shown in Fig. S4 (b). Fig. S4 (a) shows that the ${\mathbf{J}_{\mathbf{sw}}}/{H_{a\varphi}}$ increases with increasing $H_{e}$ but it becomes almost constant above ~100 T. This suggests that the strength of $H_{a\varphi}$ is more influential to $\mathbf{J}_{\mathbf{sw}}$ than the strength of the exchange field (for NiO, $H_{e}$~ 1000 T)^[[4]](#endnote-4)^. We also note that the trajectories of **m_1_** and **m_2_** (see Fig. S3 (d) ) sway slightly in z direction due to the effective field $\mathbf{J}_{\boldsymbol{s,\sigma}}\times\mathbf{m}_{\boldsymbol{\sigma}}$. This results in the bending of the antiparallel coupling between **m_1_** and **m_2_** which would cost the exchange energy. However, the maximal bending during the rotational switching shown in Fig. S4 (c) indicates that the bending is not an important factor for the switching (It only gives the bending equivalent to 30 mT effective field with $H_{e}$= 1000 T).

Assuming $H_{e}$= 1000 T ^4^, our critical current *J_sw_* = 4 x 10^7^ A/cm^2^ leads to the anisotropy field of $H_{a\varphi}$ ~ 1mT which is an order of magnitude smaller than the reported value $H_{a\varphi}$ ~ 30mT for a single crystal NiO^4^. This discrepancy may indicate the reduction of the effective anisotropy energy due to the spin rotation associated by the domain motion as well as the significant heating by the flow of the switching current (we estimated ~120 K increase with 27 mA switching current injection. See Section 4.).


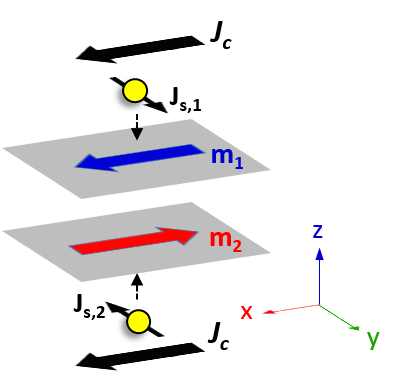


**Figure S2 The geometry of the macro-spin simulation.** The current density $\mathbf{J}_{\mathbf{c}}$ flowing in the Pt layer invokes the spin Hall effect and inject the spin current with the polarization $\mathbf{J}_{\boldsymbol{s,\sigma}}$. $\mathbf{J}_{\boldsymbol{s,\sigma}}$ exerts a spin torque on $\mathbf{m}_{\boldsymbol{\sigma}}$ which are antiferromagnetically coupled by $H_{e}$.

| **Table S1 Parameters used in the simulation** |
| --- |
| Parameters |
| Magnetic moment^[[5]](#endnote-5)^: $M_{\sigma}=0.3 T$ |
| Thickness: $t=10 nm$  Exchange field: $H_{e}=0 \sim2500 T$ |
| Easy plane anisotropy^4^: $H_{a\theta}={-2\left( K_{1}+2K_{2} \right)}/{M_{\sigma}}$= 0.6 *T* |
| 6-fold magnetic anisotropy^4^: $H_{a\varphi}={{36K}_{3}}/{M_{\sigma}=0 \sim0.05 T}$ |
| Spin Hall angle: $\theta_{SH}=0.1$ |
| Damping^[[6]](#endnote-6)^: $\alpha={10}^{-4}$ |
| 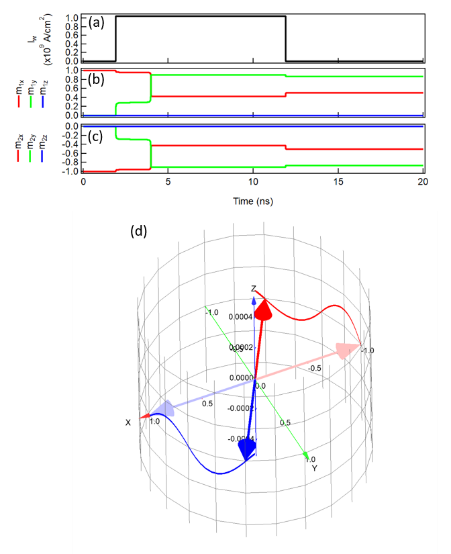  **Figure S3 Typical trajectory of** **m_1_ and m_2_ at a critical current** $\mathbf{J}_{\mathbf{sw}}$ **with** $\boldsymbol{H}_{\boldsymbol{e}}\boldsymbol{=}\boldsymbol{1000 T}$ **and** $\boldsymbol{H}_{\boldsymbol{a\varphi}}\boldsymbol{=0.03 T}$**.** (a) applied *J_w_*, (b) Cartesian components of **m_1_**_,_ and (c) Cartesian components of **m_2_** as a function of time. (d) **m_1_** and **m_2_** rotate from the initial state (**m_1_** = $+\hat{\mathbf{x}}$, **m_2_** = $-\hat{\mathbf{x}}$, light colored arrows) to one of the easy axes (**m_1_** = $1/2\hat{\mathbf{x}}{+\sqrt{3}x}/2\hat{\mathbf{y}}$, **m_2_** = ${-1}/2\hat{\mathbf{x}}{-\sqrt{3}x}/2\hat{\mathbf{y}}$, dark colored arrows). Note that the z-axis scale is magnified $2\times{10}^{3}$ times. |


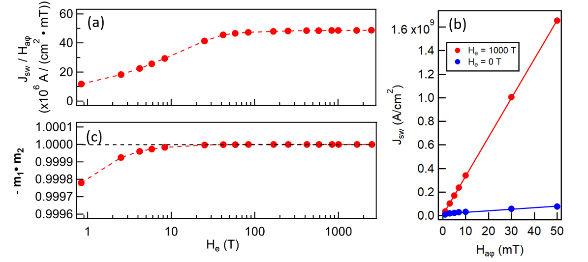


**Figure S4 Critical switching current and relative angle between m_1_ and m_2_**. (a) ${J_{sw}}/{H_{a\varphi}}$ and (b) $J_{sw}$ as a function of $H_{a\varphi}$ (c) The maximal $-\mathbf{m}_{\mathbf{1}}\boldsymbol{\cdot}\mathbf{m}_{\mathbf{2}}$ during the rotational switching as a function of $H_{e}$.

1. **Comparison of the magnitude of the spin Hall magnetoresistance (SMR)**

By adopting the formalism of the regular ferromagnetic SMR, the transverse SMR ratio defined by the transverse resistivity change $\Delta\rho_{1}$ over the resistivity $\rho$ of Pt can be written as ^[[7]](#endnote-7)^,

$$\frac{\Delta\rho_{1}}{\rho}=\theta_{SH}^{2}\frac{\lambda}{d_{N}}Re\left( \frac{2\lambda G_{\uparrow\downarrow}\tanh^{2} \frac{d_{N}}{2\lambda}}{\sigma+2\lambda G_{\uparrow\downarrow}\coth\frac{d_{N}}{\lambda}} \right) (S4)$$

where $\theta_{SH}$, $\lambda$, $d_{N}$, and $\sigma$ are the spin Hall angle, the spin diffusion length, the thickness, and the bulk conductivity of the Pt. $G_{\uparrow\downarrow}$ is the mixing conductance of the Pt/ NiO interface.

In our devices, the measured Pt resistivity $\rho$ is 6.4 x 10^-7^ Ω m and $\Delta R_{Hall} \sim110$ mΩ corresponding to $\Delta\rho_{1} \sim$4.4 x 10^-10^ Ω m, leading to ${\Delta\rho_{1}}/\rho$ ~ 7 x 10^-4^ which is comparable to the reported values ^[[8]](#endnote-8),^^[[9]](#endnote-9)^.

On the other hand, by assuming^8^ $\theta_{SH}$ = 0.1, $\lambda$ = 2.4 nm, and $G_{\uparrow\downarrow}$=10^18^ Ω^-1^ m^-2^ , Equation S4 yields ${\Delta\rho_{1}}/\rho$ ~ 3 x 10^-3^ with $d_{N}$ = 4 nm (considering only one of the interfaces of Pt/ NiO) and the conductivity of the Pt $\sigma$ = 1.6 x 10^6^ Ω^-1^ m^-1^, suggesting the experimentally obtained ${\Delta\rho_{1}}/\rho$ is well within the framework of the SMR theory.

We should note that $\Delta R_{Hall}$ may originate not only from SMR but also from other factors. One of the most likely possibilities is a non-uniform spatial distribution of the resistivity at the Hall cross part. Since the portions switched by the spin-torque are not spatially uniform, the resistance change due to SMR spatially varies. This non-uniform distribution of the resistance may give a fictitious enhancement of $\Delta R_{Hall}$.

1. **Device temperature during the writing**

It is important to know the actual device temperature while flowing the relatively large amount of the writing current. Here, we show the experimental estimation of the temperature rise due to the writing current by the device resistance. Figure S5 shows representative results of a Pt 4nm/ NiO 10nm/ Pt 4nm device. As the writing current (flowing from the 1, 2 electrodes to the 3, 4 electrodes) increases, the device resistance along the current flow increases quadratically as shown in Fig. S5 (a), indicating that the Joule heating is a dominant factor. Temperature dependence of the device resistance measured with a small bias current of 1 mA is shown in Fig. S5 (b). By referencing the temperature variation of the device resistance (Fig. S5 (b)), we estimated the device temperature rise due to the writing current flow. During our writing operations, the device temperature can rise up to ~120 K (Fig. S5 (c)). This rather significant temperature rise may effectively reduce the magnetic anisotropy and help the rotational switching.


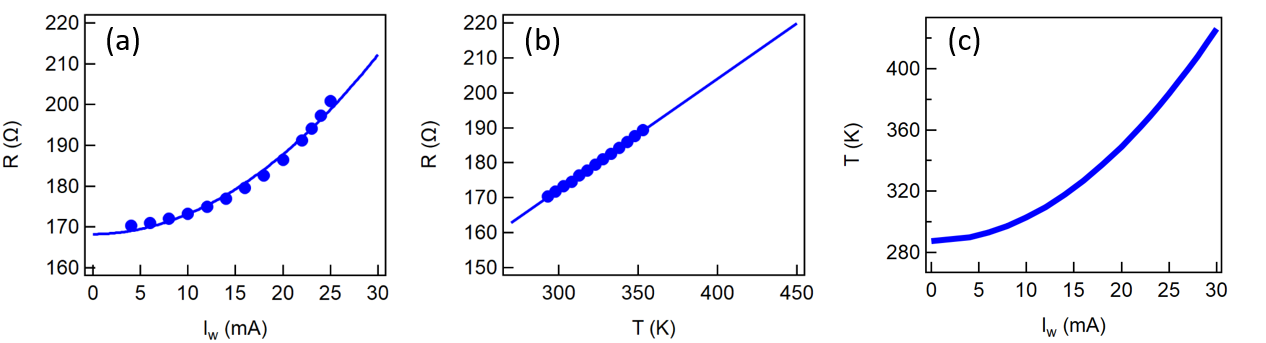


**Figure S5 Estimation of the device temperature during the writing operation.** (a) Device resistance as a function of the writing current *I_w_*. (b) Temperature dependence of the device temperature. (c) Estimated device temperature as a function of *I_w_*.

1. **Image processing**

Here, we explain how we post-processed the images of XMLD shown in Fig. 3 (e). The raw images (those shown in Fig. 3 (a) and (b)) were trimmed to the size of interest. The gray scale histogram shows two maxima corresponding to the NiO domain (the light and dark parts of the image) as shown in Fig. S6. We binned the pixels into white and black with reference to the minima of the histogram and reproduced the monochrome image (shown on the right.). With this method, we equally treated two images of the state “1” and “0”, and identify the delimiting boundaries of the NiO domains.


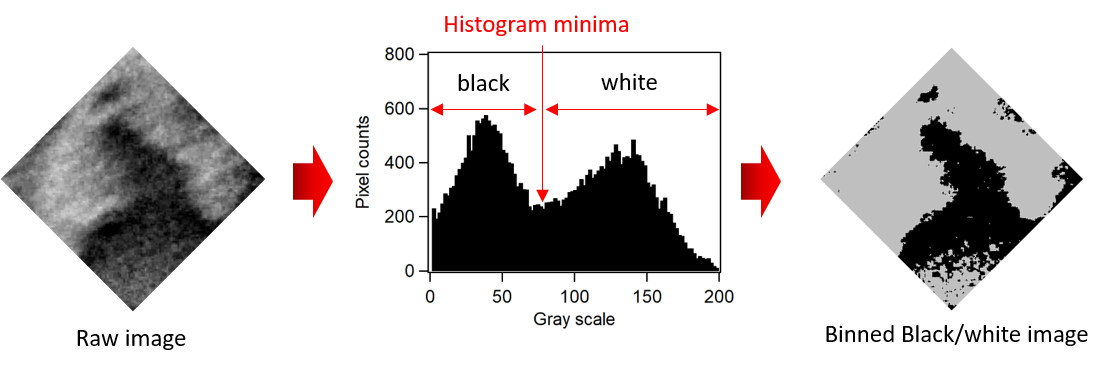


**Figure S6 Post-processing of the XMLD-PEEM images.**

**References**

1. W. L. Roth, *Phys. Rev.* **110**, 1333 (1958). [↑](#endnote-ref-1)
2. H. Wang, C. Du, P. C. Hammel, and F. Yang, *Phys. Rev. Lett.* **113**, 097202 (2014). [↑](#endnote-ref-2)
3. T. Moriyama, S. Takei, M. Nagata, Y. Yoshimura, N. Matsuzaki, T. Terashima, Y. Tserkovnyak, and T. Ono, *Appl. Phys. Lett.* **106**, 162406 (2015). [↑](#endnote-ref-3)
4. S. M. Rezende, R. L. Rodríguez-Suárez, and A. Azevedo, *Phys. Rev. B* 93, 054412 (2016). [↑](#endnote-ref-4)
5. J. Hugel and M. Kamal, *Solid State Commun.* **100**, 457 (1996). [↑](#endnote-ref-5)
6. T. Kampfrath, A. Sell, G. Klatt, A. Pashkin, S. Mährlein, T. Dekorsy, M. Wolf, M. Fiebig, A. Leitenstorfer, and R. Huber, *Nat. Photon.* **5**, 31 (2011). [↑](#endnote-ref-6)
7. Y.-T Chen, S. Takahashi, H. Nakayama, M. Althammer, S. T. B. Goennenwein, E. Saitoh, and G. E. W. Bauer, *Phys. Rev. B* **87**, 144411 (2013). [↑](#endnote-ref-7)
8. G. R. Hoogeboom, A. Aqeel, T. Kuschel, T. T. M. Palstra, and B. J. van Wees, *Appl. Phys. Lett.* **111**, 052409 (2017) [↑](#endnote-ref-8)
9. L. Baldrati, A. Ross, T. Niizeki, R. Ramos, J. Cramer, O. Gomonay, E. Saitoh, J. Sinova, M. Kläui, *arXiv:1709.00910* [↑](#endnote-ref-9)
